# A New Era in Early Postoperative OCT: Swept-Source Versus Spectral-Domain in Gas-Filled Eyes

**DOI:** 10.3390/diagnostics16152440

**Published:** 2026-08-02

**Authors:** Federico Giannuzzi, Mattia Cusato, Umberto De Vico, Diletta Paganelli, Lorenzo Hu, Giuseppe Liuzzi, Kevin Forgione, Paolo Lando, Arianna Pignatelli, Miriana Capodiferro, Valentina Cestrone, Ludovica Paris, Maria Cristina Savastano, Stanislao Rizzo

**Affiliations:** 1 Università Cattolica del Sacro Cuore, 00168 Rome, Italy; federico.giannuzzi@gmail.com (F.G.); cusato.mattia@gmail.com (M.C.); diletta.paganelli@gmail.com (D.P.); lorenzo.hu14@gmail.com (L.H.); giuseppe.liuzzi2001@gmail.com (G.L.); kevinforgione@gmail.com (K.F.); paolo.lando1997@gmail.com (P.L.); ariannapignatelliap@gmail.com (A.P.); miry.capodiferro@gmail.com (M.C.); valentinacestrone@gmail.com (V.C.); ludovica.paris@policlinicogemelli.it (L.P.); mariacristina.savastano@gmail.com (M.C.S.); stanislao.rizzo@gmail.com (S.R.); 2 Ophthalmology Unit, Fondazione Policlinico Universitario A. Gemelli, IRCCS, 00168 Rome, Italy; 3 Consiglio Nazionale delle Ricerche (CNR), Istituto di Neuroscienze, 56024 Pisa, Italy

**Keywords:** spectral-domain optical coherence tomography, swept-source optical coherence tomography, SS-OCT, SD-OCT, vitreoretinal surgery, gas-filled eyes

## Abstract

**Objectives**: This study aims to compare the imaging performance of spectral-domain optical coherence tomography (SD-OCT) and swept-source OCT (SS-OCT) in the early postoperative assessment of patients undergoing vitreoretinal surgery with intraocular gas or air tamponade. **Methods**: Seventeen eyes of 17 patients who underwent pars plana vitrectomy with either sulfur hexafluoride (SF_6_, 20%) or air tamponade were prospectively enrolled. All patients underwent OCT imaging on postoperative day 1 using both the SD-OCT system and the SS-OCT platform, without pharmacological mydriasis. Images were independently evaluated by two experienced ophthalmologists based on the ability to delineate four prespecified anatomical layers: inner retinal layers, ellipsoid zone (EZ), retinal pigment epithelium (RPE) and choroid. Images were classified as adequate quality if two or more of these structures were identifiable. **Results**: SS-OCT provided adequate-quality images in all 17 eyes (100%), with complete visualization of the inner retinal layers, EZ, RPE, and choroid in 15 eyes (88.2%). In contrast, SD-OCT yielded adequate-quality images in only three of 17 eyes (17.6%), demonstrating marked signal attenuation, interface artifacts, and inability to resolve deeper retinal structures in most cases. No difference in imaging performance was observed between SF_6_ and air tamponade subgroups. **Conclusions**: SS-OCT demonstrates markedly superior imaging performance in gas-filled eyes on postoperative day 1 compared to SD-OCT, primarily attributable to its longer wavelength, reduced sensitivity roll-off, and superior penetration through optically challenging media. These findings suggest that SS-OCT may offer meaningful advantages for early postoperative monitoring following vitreoretinal surgery with tamponade; confirmation in larger prospective cohorts with clinical-outcome correlation is warranted before it can be recommended as the preferred modality.

## 1. Introduction

Optical coherence tomography (OCT) has become an indispensable imaging modality in contemporary ophthalmology, enabling non-invasive, high-resolution, cross-sectional visualization of retinal microarchitecture. Two principal OCT technologies are currently used in clinical practice: spectral-domain OCT (SD-OCT) and swept-source OCT (SS-OCT), each with distinct optical and technical properties that confer specific advantages and limitations.

SD-OCT systems employ a broadband superluminescent diode with spectrometer-based detection, operating at a central wavelength of approximately 840 nm. This configuration permits high axial resolution (≈5–7 µm) and excellent delineation of superficial retinal microstructures but exhibits a pronounced sensitivity roll-off with increasing imaging depth [1,2,3]. For this reason, SD-OCT is limited by reduced penetration into the choroid and signal degradation in the presence of media opacities. To partially address this limitation, Enhanced Depth Imaging (EDI) techniques have been developed, improving choroidal visualization by repositioning the zero-delay line adjacent to the choroidal plane [4].

SS-OCT systems utilize a tunable swept laser source operating at longer wavelengths (~1050 nm) with photodetector-based acquisition. This architecture allows several advantages: higher scanning speeds, reduced sensitivity roll-off, and improved depth penetration, resulting in superior visualization of posterior ocular structures, particularly the choroid, and enhanced performance in optically challenging conditions [5,6,7].

The technical characteristics of both technologies are available in Table S1 in the Supplemental Materials.

The comparative performance of SD-OCT and SS-OCT has been extensively investigated in multiple clinical contexts, including choroidal thickness measurement, visualization of choroidal neovascularization, assessment of the optic nerve head, and imaging in conditions associated with media opacity. These studies have collectively demonstrated that SS-OCT brings meaningful advantages in scenarios where signal attenuation through anatomical or pathological barriers is a limiting factor, thanks to its longer wavelength and improved depth penetration [8,9,10,11,12,13,14,15].

The early postoperative period following vitreoretinal surgery with intraocular gas or air tamponade represents one such challenging scenario. Gas and air within the vitreous cavity introduce substantial refractive index (RI) mismatches at the gas–tissue interface (RI air ≈ 1.00 vs. RI tissue ≈ 1.33–1.40), generating Fresnel reflections, signal attenuation, and interfacial artifacts that severely degrade image quality [16]. Nevertheless, OCT imaging during this period is clinically important: it enables the monitoring of macular status, assessment of subretinal fluid reabsorption in retinal detachment, confirmation of epiretinal membrane removal, and evaluation of foveal anatomy following macular hole repair.

Despite the recognized difficulty of OCT imaging in gas-filled eyes, head-to-head comparisons between SD-OCT and SS-OCT in this specific clinical context remain limited. The present study was designed to directly compare the imaging performance of SD-OCT and SS-OCT in patients on postoperative day 1 following vitreoretinal surgery with tamponade, using predefined qualitative image quality criteria. Although the superior penetration of SS-OCT through optically challenging media is well established, its incremental value specifically on postoperative day 1, the earliest and most artifact-prone window, when management decisions such as face-down positioning and detection of persistent subretinal fluid are made, has not been quantified in a prospective head-to-head design. Accordingly, the specific objectives of this study were, in order: (i) to compare image-acquisition success; (ii) to compare the delineation of four prespecified retinal structures; and (iii) to compare overall clinical interpretability between SD-OCT and SS-OCT in gas- or air-filled eyes on postoperative day 1 [17].

## 2. Materials and Methods

### 2.1. Study Design and Participants

This prospective observational study enrolled 17 consecutive eyes of 17 patients who underwent pars plana vitrectomy (PPV) at the Ophthalmology Unit, Fondazione Policlinico Universitario A. Gemelli-IRCCS, Rome, Italy. During the study period, 30 eyes underwent pars plana vitrectomy at our institution. Seven eyes were not eligible because surgery was concluded with silicone oil (*n* = 5) or balanced salt solution (*n* = 2) rather than gas or air tamponade. Of the 23 eyes assessed for eligibility, 5 were excluded: 3 because corneal or lenticular opacity precluded OCT acquisition, and 2 because the tamponade occupied less than 50% of the vitreous cavity or did not cover the macular area at the time of imaging. Eighteen eyes were enrolled after informed consent, of which one did not undergo imaging on postoperative day 1. The final analysis therefore comprised 17 eyes of 17 patients, all of which were imaged with both devices on postoperative day 1.

Surgical indications included epiretinal membrane, macular hole, rhegmatogenous retinal detachment, and silicone oil removal. All patients enrolled in the study were pseudophakic. The study was conducted in accordance with the tenets of the Declaration of Helsinki. Informed consent was obtained from all participants.

Patients were eligible for inclusion if they had undergone PPV with air or 20% SF_6_ gas tamponade, were available for OCT imaging on postoperative day 1, had clear anterior segment media permitting fundus examination, and had a vitreous cavity filled by air or gas to more than 50% of its volume at the time of imaging with tamponade covering the macular area. Patients were excluded if they had received BSS or silicone oil tamponade, or had significant corneal or lenticular opacity precluding OCT acquisition.

**Figure 1:**
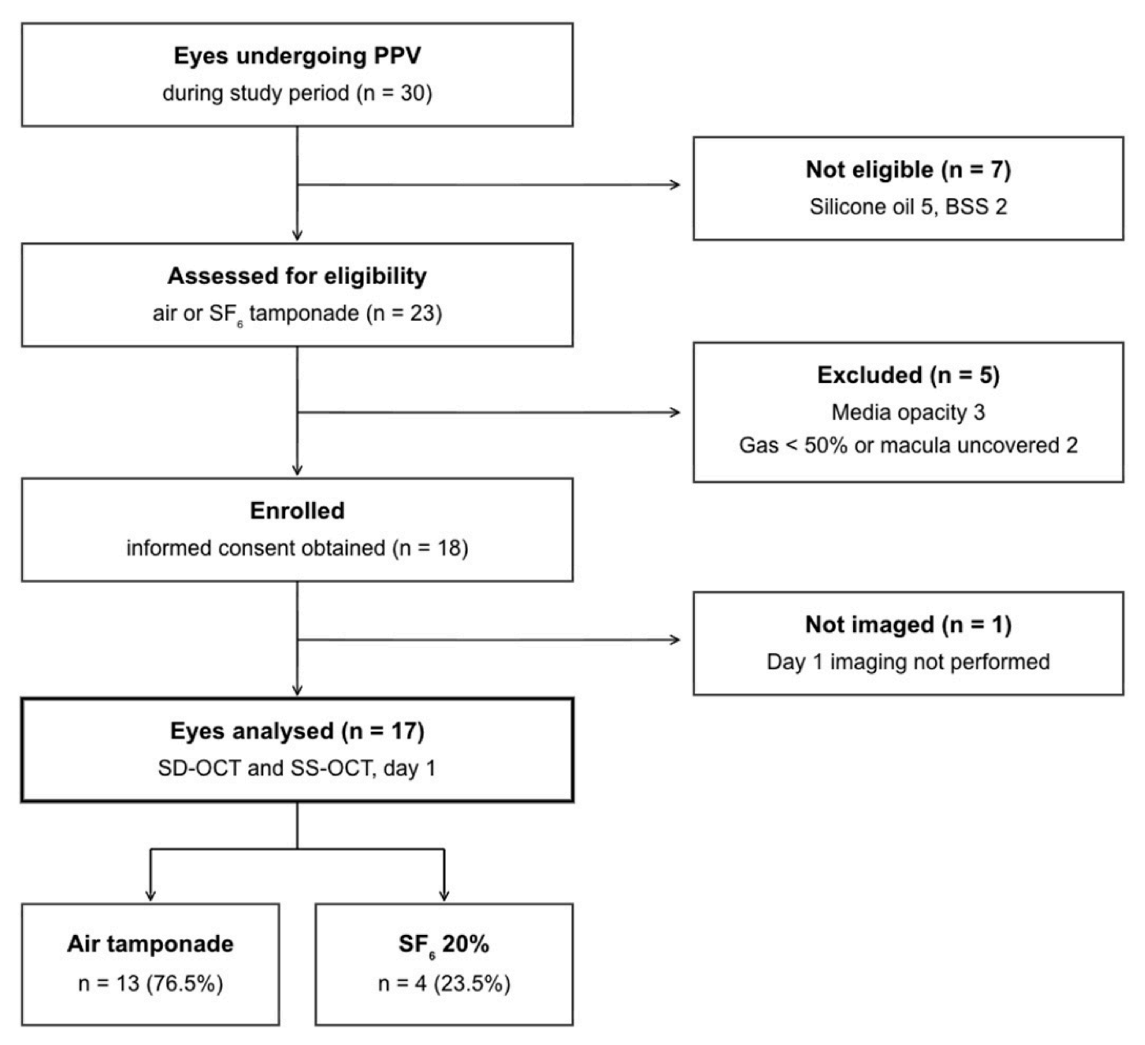
Flow diagram showing patient inclusion and exclusion criteria.

The selection process is summarized in Figure 1.

### 2.2. Imaging Devices

Imaging was performed using two devices. SD-OCT was acquired with a 120 k-Hz system and SS-OCT with a 400 kHz platform. Spectral-Domain OCT (SD-OCT) was performed with the Solix system (Optovue Inc., Fremont, CA, USA), whereas Swept-Source OCT (SS-OCT) was performed with the TowardPi BMizar 400 kHz OCT system (TowardPi Medical Technology Co., Beijing, China). Technical specifications of both devices are summarized in Table 1.

### 2.3. Imaging Protocol

All patients underwent OCT imaging within 24 h of surgery, in a state of pharmacological miosis (without mydriatic drops), performed by three experienced ophthalmologists. The scanning protocol included 12 mm and 24 mm horizontal and vertical linear B-scans, as well as raster scan acquisitions, using both devices. SS-OCT was performed prior to SD-OCT in 9 eyes, while the opposite sequence (SD-OCT followed by SS-OCT) was applied in 8 eyes.

### 2.4. Image Analysis

Images were evaluated independently by two experienced ophthalmologists (M.C. and D.P.). The evaluation was based on predefined parameters: (i) success rate of image acquisition, defined as the proportion of eyes in which an evaluable macula-centered B-scan could be obtained with each device, irrespective of subsequent quality grading, and (ii) ability to delineate four anatomical structures: inner retinal layers, ellipsoid zone (EZ), retinal pigment epithelium (RPE), and choroid. Images were classified as adequate quality if two or more of these structures were clearly identifiable; images in which fewer than two structures could be delineated were classified as inadequate quality. In cases of disagreement, a third senior ophthalmologist adjudicated to reach consensus. This two-structure threshold was chosen a priori to reflect the minimum information required for clinically meaningful postoperative interpretation, identification of at least the retinal plane and the RPE/outer-retina complex, rather than any previously validated index; we acknowledge this operational definition as a study-specific choice (see Limitations). Inter-rater agreement between the two independent graders was excellent (Cohen’s κ = 0.91), consistent with a high degree of concordance in image quality classification.

### 2.5. Statistical Analysis

All statistical analyses were performed using STATA17. Categorical variables are reported as absolute frequencies and percentages. The primary outcome, adequacy of image quality, was compared between SD-OCT and SS-OCT using the McNemar test for paired proportions, given the within-subject design in which both devices were applied to the same eyes. A *p*-value of less than 0.05 was considered statistically significant. Inter-rater agreement for image quality classification was assessed using Cohen’s kappa coefficient (κ), with values interpreted according to the Landis and Koch scale: κ > 0.80 was considered to represent almost perfect agreement.

## 3. Results

### 3.1. Patient Characteristics

Seventeen eyes of 17 patients were included. The distribution of surgical indications was as follows: macular hole (*n* = 5, 29.4%), epiretinal membrane (*n* = 6, 35.3%), rhegmatogenous retinal detachment (*n* = 2, 11.8%), and silicone oil removal (*n* = 4, 23.5%). Intraocular tamponade consisted of SF_6_ 20% in four eyes (23.5%) and air in 13 eyes (76.5%). Among the enrolled patients, seven were female (41.2%) and 10 were male (58.8%). On the postoperative day 1, all 17 eyes had tamponade occupying more than 50% of the vitreous cavity, with the macular area fully covered by gas or air in all cases.

### 3.2. Imaging Performance

Of the 17 eyes examined with SD-OCT, only three (17.6%) produced images of sufficient quality for evaluation. Among these, just one eye (5.9%) allowed clear visualization of the inner retinal layers, the RPE, and the choroid. In the remaining two eyes (11.8%), the RPE signal could be identified; however, reliable delineation of the inner retinal layers and the ellipsoid zone was not possible, and choroidal visualization remained limited. In the other 14 eyes (82.4%), image quality was inadequate for clinical interpretation, preventing reliable identification of both the retinal plane and the RPE. No statistically or clinically meaningful differences in SD-OCT performance were observed between eyes treated with SF_6_ and those receiving air tamponade.

In contrast, all 17 eyes (100%) examined with SS-OCT yielded images of adequate quality. Complete visualization of the inner retinal layers, ellipsoid zone, RPE, and choroid was achieved in 15 eyes (88.2%). In the remaining two eyes (11.8%), one filled with air and one with SF_6_,only the RPE could be consistently identified, whereas the inner retinal layers, ellipsoid zone, and choroid were not clearly distinguishable. En face imaging was not interpretable in any of the 17 eyes because of gas- and air-related artifacts. Mild diffraction artifacts were noted in the B-scans of four eyes (23.5%); however, these did not significantly affect the visualization of retinal structures.

The qualitative imaging outcomes for both devices are summarized in Table 2.

All images are available as Supplemental Materials in the link.

SS-OCT demonstrated statistically superior imaging performance compared to SD-OCT across all primary outcome measures. The rate of adequate image quality was significantly higher with SS-OCT than with SD-OCT (SS-OCT 100% [95% CI 80.5–100.0%] vs. SD-OCT 17.6% [95% CI 3.8–43.4%]; *p* < 0.001, McNemar exact test), as was the rate of full visualization of all four prespecified retinal structures (88.2% [95% CI 63.6–98.5%] vs. 5.9% [95% CI 0.1–28.7%]; *p* < 0.001). Conversely, the proportion of eyes yielding inadequate images was significantly lower with SS-OCT than with SD-OCT (0% vs. 82.4%; *p* < 0.001). The proportion of eyes in which only the RPE was identifiable did not differ significantly between the two devices (11.8% vs. 11.8%; *p* = 1.000). No statistically significant difference in imaging performance was observed between eyes treated with SF_6_ and those treated with air tamponade for either device (*p* = 1.000, Fisher exact test).

**Figure 2:**
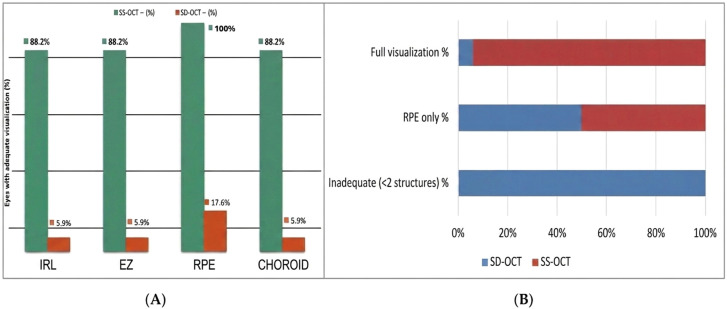
Comparison of retinal structure visualization between SS-OCT and SD-OCT in 17 gas-filled eyes on postoperative day 1. (**A**) Percentage of eyes with adequate visualization of each prespecified retinal structure: inner retinal layers (IRL), ellipsoid zone (EZ), retinal pigment epithelium (RPE), and choroid. SS-OCT achieved full structural visualization in 88.2–100% of eyes across all layers, compared to 5.9–17.6% with SD-OCT. (**B**) Distribution of image quality outcomes. SD-OCT yielded inadequate images in 82.4% of eyes; SS-OCT provided adequate images in 100% of eyes. IRL, inner retinal layers; EZ, ellipsoid zone; RPE, retinal pigment epithelium; SD-OCT, spectral-domain optical coherence tomography; SS-OCT, swept-source optical coherence tomography.

**Figure 3:**
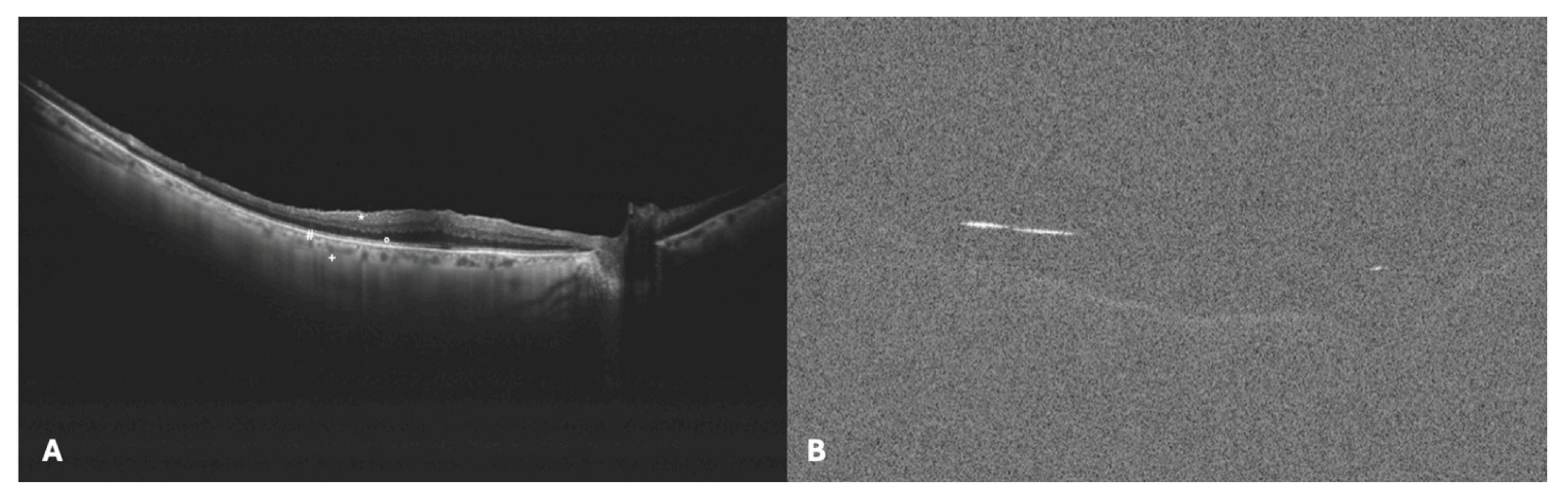
Comparison between images acquired with SS-OCT (**A**) and SD-OCT (**B**) in a patient following pars plana vitrectomy (PPV) for an epiretinal membrane with air tamponade. In Figure 2A inner retinal layers (*), EZ (°), RPE (#) and choroid (+) are visible.

**Figure 4:**
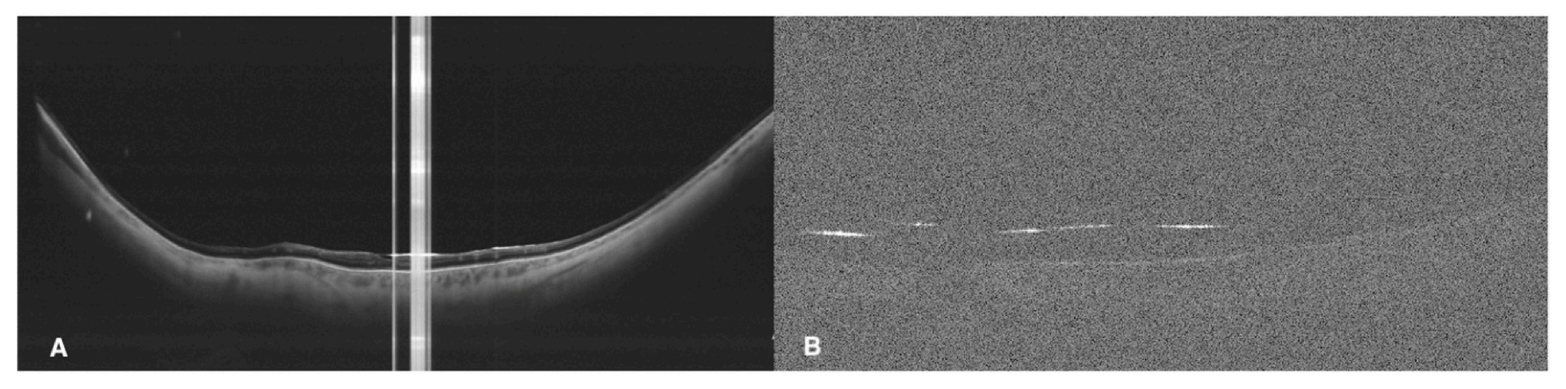
Comparison between images acquired with SS-OCT (**A**) and SD-OCT (**B**) in a patient following pars plana vitrectomy (PPV) for macular hole with SF_6_ tamponade. In (**A**) mild diffraction artifacts were noted in the B-scan; however, these did not significantly affect the visualization of retinal structures.

**Figure 5:**
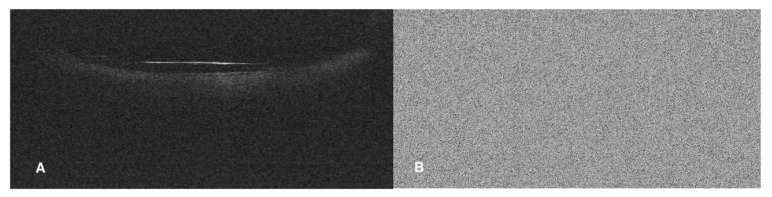
Comparison between images acquired with SS-OCT (**A**) and SD-OCT (**B**) in a patient following pars plana vitrectomy (PPV) for epiretinal membrane with air tamponade. In (**A**) only the RPE could be consistently identified, whereas the inner retinal layers, ellipsoid zone, and choroid were not clearly distinguishable.

Figure 2 summarizes the results of the comparison between SD-OCT and SS-OCT in terms of retinal structure visualization quality, while Figure 3, Figure 4 and Figure 5 shows the difference between the images obtained with both technologies in the same patient at the same time.

## 4. Discussion

The principal finding of this study is that SS-OCT achieves high-quality retinal imaging in all gas-filled eyes on postoperative day 1. In contrast, SD-OCT provides adequate image quality in fewer than one-fifth of cases under the same conditions. These results confirm and extend the known technological advantages of SS-OCT in optically challenging environments and have direct implications for postoperative clinical management following vitreoretinal surgery with tamponade.

The inferior performance of SD-OCT in gas-filled eyes can be attributed to several interacting optical mechanisms. The refractive index mismatch at the gas–tissue interface (RI air ≈ 1.00 vs. RI tissue ≈ 1.33–1.40) generates strong Fresnel reflections and refraction, reducing the transmitted optical power and introducing interface artifacts that distort axial localization [16]. Gas inclusions and microbubbles act as highly reflective boundaries, generating specular reflections that may saturate the spectrometer detector and mask adjacent structural information. Furthermore, multiple scattering events within inhomogeneous gas regions redistribute photons into incoherent paths, reducing fringe visibility and degrading the interferometric signal required for Fourier-domain reconstruction. These effects are compounded by the greater sensitivity roll-off inherent to SD-OCT at the shorter wavelength of 840 nm, which limits the effective imaging depth and signal-to-noise ratio in the presence of any attenuating medium.

In contrast, SS-OCT mitigates these limitations through several complementary mechanisms: (i) its longer operating wavelength (~1050 nm) is less susceptible to scattering by gas–tissue interfaces; (ii) its higher scanning speed (up to 400,000 A-scans/s) reduces motion artifacts and allows averaging over more A-scans; (iii) its reduced sensitivity roll-off maintains signal quality over a greater imaging depth; and (iv) its deeper maximum imaging range accommodates the altered optical geometry of a gas-filled globe. Collectively, these properties enable consistent penetration through the gas–vitreous interface and reliable delineation of the neurosensory retina, RPE, and choroid.

Our findings are consistent with prior investigations demonstrating Enhanced Depth Imaging and superior choroidal visualization with longer-wavelength OCT systems [4,9,11,18]. Previous studies specifically addressing OCT in gas-filled eyes have documented the challenges faced by SD-OCT in this context, and limited comparative data have suggested a potential advantage of SS-OCT [16,19,20]. The present study provides, to our knowledge, among the largest specifically in the immediate postoperative period, using standardized imaging protocols and predefined qualitative endpoints.

From a clinical standpoint, reliable visualization of the outer retina and RPE in the immediate postoperative period is critical for several reasons. In macular hole surgery, confirmation of hole closure and assessment of foveal anatomical restoration on day 1 may influence immediate postoperative management decisions, including the duration and stringency of face-down positioning. In retinal detachment, detection of persistent subretinal fluid at the macula is important for prognostic counseling. In epiretinal membrane surgery, complete membrane removal can be verified and residual tractional features identified. In all these scenarios, SS-OCT appears to provide substantially more clinically actionable information than SD-OCT on postoperative day 1 [17,28].

It is important to note that no significant difference in SS-OCT imaging performance was observed between eyes with SF_6_ and air tamponade, suggesting that the advantages of SS-OCT extend consistently across tamponade types. In contrast, SD-OCT also showed no clear differential performance between tamponade types, reflecting uniformly poor performance in both subgroups. However, because only four eyes received SF_6_, the absence of a significant SF_6_-versus-air difference should not be read as evidence of equivalence between tamponade types; this comparison is underpowered.

Several limitations of the present study warrant acknowledgment. First, the sample size of 17 eyes is relatively small, and replication in a larger cohort is needed to confirm these findings and to enable subgroup analyses by surgical indication and tamponade type. Second, imaging was performed at a single time point (postoperative day 1), and longitudinal assessment across the postoperative course was not performed; the trajectory of image quality improvement as gas reabsorbs remains to be characterized. Third, this study assessed imaging performance only; it did not test whether the superior visualization afforded by SS-OCT altered diagnosis, management decisions, or clinical outcomes, and this correlation should be the focus of future work. Fourth, the image quality classification system used was semi-quantitative and grader-dependent, despite the use of predefined criteria and adjudication for discordant assessments; future studies should incorporate objective, quantitative image quality metrics. Fifth, en face imaging was not interpretable in any eye for either device, which limits conclusions regarding OCTA performance in gas-filled eyes. Moreover, a quantitative gas-fill measurement could be useful because a different level of tamponade could bring a different image quality. Finally, the present study compared a single SD-OCT device against a single SS-OCT device, and the results may not generalize to all SD-OCT or SS-OCT platforms.

While en face imaging was not interpretable in this study, future research should investigate the utility of widefield SS-OCT, capable of single-acquisition scans up to 24 × 20 mm, for mapping the peripheral retina through gas.

## 5. Conclusions

This prospective comparison demonstrates that SS-OCT achieves markedly superior retinal imaging in gas-filled eyes on postoperative day 1 relative to SD-OCT, providing adequate-quality images in 100% versus 17.6% of eyes, respectively. The enhanced penetration, reduced sensitivity roll-off, and greater resilience to gas–tissue interface artifacts afforded by SS-OCT make it particularly suitable for early postoperative evaluation following vitreoretinal surgery with tamponade. These findings suggest that SS-OCT may be advantageous in this clinical setting and have implications for postoperative protocols and device selection in vitreoretinal surgical units.

## Figures and Tables

**Table 1:** Technical specifications of the SD-OCT and SS-OCT systems.

Feature	SD-OCT	SS-OCT
**OCT Type**	Solix system (Optovue Inc., Fremont, CA, USA)	TowardPi BMizar system (TowardPi Medical Technology Co., Beijing, China)
**Central Wavelength, nm**	~840	~1060
**Scan Speed, A-scans/s, kHz**	Up to 120,000	Up to 400,000

Data based on manufacturer datasheets and published specifications [6,18,19,20,21,22,23,24,25,26,27].

**Table 2:** Summary of image quality outcomes for SD-OCT and SS-OCT on postoperative day 1 in 17 gas-filled eyes. Outcomes are stratified according to the number of prespecified retinal structures identifiable on B-scan imaging: full visualization (inner retinal layers, ellipsoid zone, retinal pigment epithelium, and choroid); RPE only; or inadequate quality (fewer than two structures identifiable). *P*-values were calculated using the McNemar exact test for paired proportions. RPE, retinal pigment epithelium; SD-OCT, spectral-domain optical coherence tomography; SS-OCT, swept-source optical coherence tomography.

Outcome	SD-OCT	SS-OCT	*p*-Value
**Adequate image quality**	3/17 (17.6%) [95% CI 3.8–43.4%]	17/17 (100%). [95% CI 80.5–100.0%]	<0.001
**Full visualization (all 4 layers)**	1/17 (5.9%) [95% CI 0.1–28.7%]	15/17 (88.2%) [95% CI 63.6–98.5%]	<0.001
**RPE only**	2/17 (11.8%)	2/17 (11.8%)	1.000
**Inadequate (<2 structures)**	14/17 (82.4%)	0/17 (0%)	<0.001
**Enface interpretable**	0/17	0/17	—

## Data Availability

The original contributions presented in this study are included in the article/Supplementary Material. Further inquiries can be directed to the corresponding author.

## Supplementary Materials

The following supporting information can be downloaded at: https://www.mdpi.com/article/10.3390/diagnostics16152440/s1, Table S1: Technical characteristics of SD-OCT and SS-OCT technologies. All images are available in https://drive.google.com/drive/folders/1lm-1_pmCIRhElc5OpDHYZjn9-_nlqwD_.

## Abbreviations

The following abbreviations are used in this manuscript:

SD-OCT	Spectral-domain optical coherence tomography
SS-OCT	Swept-source optical coherence tomography
EZ	Ellipsoid zone
RPE	Retinal pigment epithelium
OCT	Optical coherence tomography
EDI	Enhanced Depth Imaging
PPV	Pars plana vitrectomy
SF_6_	Sulfur hexafluoride
