# Supplementary material for: A New Era in Early Postoperative OCT: Swept-Source Versus Spectral-Domain in Gas-Filled Eyes
Source: Diagnostics (Basel). 2026 Aug 2;16(15):2440. doi: 10.3390/diagnostics16152440 (PMC13464097; doi:10.3390/diagnostics16152440)
Supplement: Supplementary file 1 [file diagnostics-16-02440-s001.zip › diagnostics-4392307-supplementary Table S1.pdf]

Table S1. Technical characteristics of SD-OCT and SS-OCT technologies.

Parameter	SD-OCT	SS-OCT
**Light Source**	Broadband superluminescent diode	Tunable swept laser
**Central Wavelength**	~840 nm	~1060 nm
**Detection Method**	Spectrometer-based	Photodetector (frequency-swept)
**Axial Resolution**	≈5–7 μm	≤5 μm
**Transverse Resolution**	14–20 μm	~10–20 μm
**Scanning Speed**	27,000–120,000 A-scans/s	100,000–400,000 A-scans/s
**Sensitivity Roll-off with Depth**	More pronounced	Reduced
**Penetration Depth**	Limited	Greater (better through media opacities)
**Signal-to-Noise Ratio at Depth**	Decreases rapidly	Better maintained
**Choroidal Visualization**	Limited; improved with EDI technique	Enhanced
**Clinical Strengths**	High-resolution retinal layer imaging	Deep imaging (choroid, postoperative eyes)
**Limitations**	Poor performance in dense or turbid media	Slightly lower axial resolution; higher cost

Note: EDI, Enhanced Depth Imaging. Data compiled from manufacturer specifications and the published literature [21].
